# Epidemiology of Seasonal Coronaviruses: Establishing the Context for the Emergence of Coronavirus Disease 2019

**DOI:** 10.1093/infdis/jiaa185

**Published:** 2020-04-14

**Authors:** Sema Nickbakhsh, Antonia Ho, Diogo F P Marques, Jim McMenamin, Rory N Gunson, Pablo R Murcia

**Affiliations:** 1 MRC–University of Glasgow Centre for Virus Research, Institute of Infection, Immunity and Inflammation, College of Medical, Veterinary and Life Sciences, University of Glasgow, Glasgow, United Kingdom; 2 Public Health Scotland, NHS National Services Scotland, Glasgow, United Kingdom; 3 West of Scotland Specialist Virology Centre, NHS Greater Glasgow and Clyde, Glasgow, United Kingdom

**Keywords:** SARS-CoV-2, Acute respiratory infections, Disease surveillance, Coinfection, Virus-virus interactions

## Abstract

Public health preparedness for coronavirus (CoV) disease 2019 (COVID-19) is challenging in the absence of setting-specific epidemiological data. Here we describe the epidemiology of seasonal CoVs (sCoVs) and other cocirculating viruses in the West of Scotland, United Kingdom. We analyzed routine diagnostic data for >70 000 episodes of respiratory illness tested molecularly for multiple respiratory viruses between 2005 and 2017. Statistical associations with patient age and sex differed between CoV-229E, CoV-OC43, and CoV-NL63. Furthermore, the timing and magnitude of sCoV outbreaks did not occur concurrently, and coinfections were not reported. With respect to other cocirculating respiratory viruses, we found evidence of positive, rather than negative, interactions with sCoVs. These findings highlight the importance of considering cocirculating viruses in the differential diagnosis of COVID-19. Further work is needed to establish the occurrence/degree of cross-protective immunity conferred across sCoVs and with COVID-19, as well as the role of viral coinfection in COVID-19 disease severity.

## Teaser

Our study provides a comprehensive evaluation of in whom, and when, seasonal coronavirus infections occur. We compare infection patterns across coronavirus types, evaluate evidence of interactions with other respiratory viruses, and discuss implications for newly emerged coronavirus disease 2019.

**(See the Major Article by Monto et al., on pages 9–16.)**

In March 2020, the World Health Organization declared the global spread of coronavirus (CoV) disease 2019 (COVID-19), caused by a human CoV (severe acute respiratory syndrome CoV [SARS-CoV-2]) that emerged in China in December 2019, a pandemic [1]. Predicting the public health impact of pathogens with recently acquired human-to-human transmissibility is a challenge. Currently, the fate of COVID-19 remains unclear; understanding the likely age and seasonal profiles of infection risks will be critical to inform effective surveillance and control strategies.

During the early phase of an outbreak, in the absence of detailed country-specific knowledge, preliminary risk estimates may be gauged from endemic pathogens with similar modes of transmission. The infection incidence and levels of severe illness associated with COVID-19 remains unclear. In this instance, epidemiological data on seasonal CoVs (sCoVs) may provide valuable information about individuals and seasonal conditions favoured by, or limiting, an invading CoV.

To date, emergent zoonotic human CoVs associated with high case-fatality ratios have not achieved persistence in the human population. SARS-CoV emerged in 2002 and spread rapidly around the globe before being successfully contained in 2003 [2]. Conversely, Middle East respiratory syndrome CoV has continued to cause sporadic cases predominantly in healthcare settings since its discovery in 2012, but has not demonstrated sustained community transmission [3]. In contrast, CoV-229E, CoV-NL63, CoV-OC43, and CoV-HKU1 are common cocirculating sCoVs predominantly associated with mild infection of the upper respiratory tract [4].

A key determinant governing the invasion and persistence success of a new pathogen is the abundance of susceptible hosts. Such population susceptibility may be difficult to define owing to preexisting cross-protective immunity in individuals previously exposed to antigenically related pathogens, as demonstrated for pandemic influenza A H1N1 in 2009 [5]. Furthermore, the potential for heterologous interactions among taxonomically broad groups of respiratory viruses is also recognized [6–11]. A good epidemiological understanding of cocirculating viruses will provide valuable information on the potential for immune, or otherwise mediated, virus-virus interactions and the consequences for population susceptibility.

To date, epidemiological knowledge surrounding sCoVs has been limited for many settings owing to their historic association with mild illness. However, some laboratories have adopted sCoV testing as part of routine multiplex diagnostic screens [12–15], following an increased recognition of the associated disease spectrum. Our group previously reported on the comparative epidemiological characteristics of acute viral respiratory infections, and the potential for virus-virus interactions, based on multiplex reverse-transcription polymerase chain reaction (PCR) testing in the West of Scotland [6, 16]. In the current article, we provide further detail on sCoVs differentiated at the species level (sCoV types) over an extended time frame and discuss key potential implications for COVID-19 virus emergence in Scotland, United Kingdom.

## METHODS

### The Study Population

Routine molecular testing for CoV-229E, CoV-OC43, and CoV-NL63 using multiplex real-time reverse-transcription PCR methods was conducted between 1 January 2005 and 30 September 2017 by the West of Scotland Specialist Virology Centre in NHS Greater Glasgow and Clyde, the largest Scottish National Health Service (NHS) board serving a population of approximately 1.2 million [17]. The respiratory virus screen also simultaneously detected influenza A virus, influenza B virus, respiratory syncytial virus (RSV), human adenoviruses (AdVs), human rhinoviruses, human metapneumovirus, and parainfluenza virus (PIV) types 1–4. The CoV-HKU1 assay was discontinued in 2012 owing to low levels of detection. Most clinical specimens (91%) were obtained from the upper respiratory tract (the majority being nasal and/or throat swab samples).

**Figure 1.**
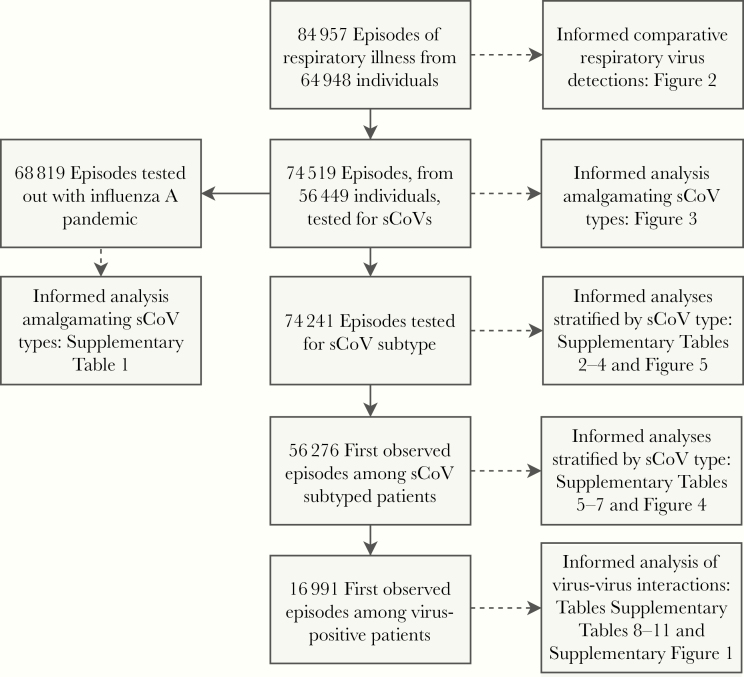
Data flow diagram summarizing patient subsets informing each analysis. Samples from 64 948 patients were subjected to molecular tested for respiratory viruses, performed with real-time multiplex reverse-transcription polymerase chain reaction in NHS Greater Glasgow and Clyde, Scotland, United Kingdom, between 1 January 2005 and 30 September 2017. Abbreviation: sCoV, seasonal coronavirus.

During the study period, 107 174 clinical respiratory samples, from 64 948 individual patients, were received by the West of Scotland Specialist Virology Centre for testing. For patients with ≥2 samples submitted (24.5% of patients), the PCR test data were aggregated into individual episodes, defined as a 30-day period from the collection date of the first sample. This generated 84 957 episodes of respiratory illness for analysis. Most episodes, 93% that occurred out with the 3 major waves of pandemic influenza A(H1N1)pdm09 virus circulation in the United Kingdom (summer 2009 and influenza seasons of 2009–2010 and 2010–2011), were tested for all 11 groups of respiratory virus. Of 84 957 episodes of respiratory illness, 10 438 were not tested for CoV (98% during the 3 major waves of pandemic influenza) and thus were excluded from analyses centered on sCoVs [18]. Among the remaining 74 519 episodes of illness, another 278 were either tested for CoV-HKU1 or the CoV was untyped; these episodes were excluded from analyses differentiating sCoV type. See Figure 1 for a summary of the data subsets.

### Statistical Modeling Analyses

Of 74 241 patient episodes of respiratory illness with sCoV subtyping, 8912 patients experienced multiple episodes over the study time frame. In such cases, we retained the first observed episode to remove patient-level clustering, leaving 56 276 patient observations for analysis (Figure 1). We used multivariable logistic regression to investigate associations between sCoV types (CoV-229E, CoV-OC43, and CoV-NL63) and patient age (categorical), sex (binary), healthcare service setting (binary; primary or secondary or tertiary services), time period with respect to the 3 major waves of pandemic influenza in the United Kingdom (categorical; prepandemic, January 2005 to April 2009; pandemic, May 2009 to February 2011; and postpandemic, March 2011 to September 2017) and season (categorical). Statistical interactions between patient covariates and healthcare service setting were assessed. An α level of 5% was used to determine statistical significance of all model coefficients. The fitted models, incorporating age-healthcare service interactions, were used to generate average predicted probabilities of virus detection by age and healthcare setting.

In addition, we used multivariable logistic regression to investigate interactions between each sCoV and other groups of respiratory viruses at the within-host scale. These analyses were based on 16 991 virus-positive episodes of respiratory illness, retaining the first observed episode of illness for patients with multiple episodes. Virus-negative patients were excluded to eliminate the influence of Berkson bias, which may lead to spurious inference of disease-disease associations when these are estimated from routine healthcare data [19]. Specifically, these analyses tested whether the odds of a given virus (“exposed”) coinfecting with a given sCoV differed from the average odds among the remaining groups of viruses (“nonexposed”), thereby assessing nonrandom mixing among the virus population.

Three models were fitted, one each for CoV-229E, CoV-OC43, and CoV-NL63 (response variables). The analyses adjusted for patient age, sex, healthcare service setting, time period with respect to pandemic influenza (as described above), and the monthly background prevalence of the sCoV (response variable) to eliminate spurious virus-virus associations owing to unrelated sources of seasonality. Holm’s method was used to correct *P* values for multiple comparisons (10 virus-virus interaction hypotheses per model) [20].

All analyses were conducted using R software version 3.4.4 [21]. Logistic regression modeling was conducted using the “glm” function, and predicted probabilities were computed using “ggaverage” from the “ggeffects” package [22].

## RESULTS

### Prevalence of sCoVs Among People With Respiratory Illness

Among 84 957 episodes of respiratory illness, 79.0% were sampled at secondary or tertiary healthcare services (hospital inpatients and outpatients), and 21.0% from primary healthcare services (general practice [GP]). The sex distribution was approximately equal, with 51.6% of patients female, and the median age was 33.1 years (interquartile range, 5.6–59.1 years).

**Figure 2.**
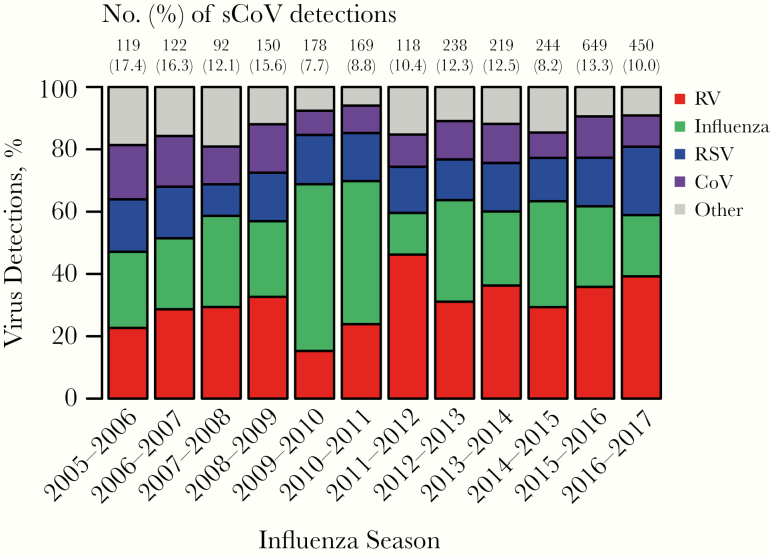
Percentages of viral respiratory infections attributed to human coronaviruses and other common respiratory viruses during each influenza season (October–May) from 2005–2006 until 2016–2017, based on 84 957 episodes of respiratory illness. Influenza includes influenza A and influenza B viruses combined; and “other” includes human adenoviruses, human metapneumovirus, and parainfluenza viruses type 1–4. Note: Years of major pandemic influenza A H1N1 virus circulation (2009–2010 and 2010–2011) must be viewed with caution, owing to high levels of partial testing. Testing for CoV-HKU1was discontinued in 2012. Abbreviations: CoV, human coronaviruses (CoV-229E, CoV-OC43, CoV-NL63, and CoV-HKU1 combined); RSV, respiratory syncytial virus; RV, human rhinovirus.

The prevalence of sCoV detections overall was 4.0% among tested patients (2958 of 74 519), contributing to 10.7% (2958 of 27 734) of all respiratory virus detections. Figure 2 summarizes the contribution of sCoVs to the total viral detections in the patient population during each influenza season (October–May) from 2005 to 2016. The most common virus detections during influenza seasons among virus-positive patients were human rhinoviruses (range, 15.3%–46.2%), influenza viruses (13.4%–34.0%, excluding pandemic influenza waves of 2009–2010 and 2010–2011), and RSV (10.1%–21.9%), followed by sCoVs (7.7%–7.4%) (Figure 2).

Numbers of sCoV detections increased before pandemic influenza (March 2011 to September 2017), likely owing to enhanced virological testing of acute respiratory illnesses; the overall number of sCoV detections rose from 545 before, to 2072 following the pandemic influenza period. However, a decrease in prevalence among the tested population was observed, from 4.27% to 3.70%, and with varying patterns at the individual sCoV level (Supplementary Table 1). The most prevalent detection was CoV-OC43, both before and following the pandemic influenza period (Supplementary Table 1). CoV-HKU1 was present at a very low prevalence of 0.3% overall (124 of 36 652 episodes tested until the assay was discontinued in 2012) and was therefore excluded from further analyses.

### Difference Between Patients in Detection of sCoVs

**Figure 3.**
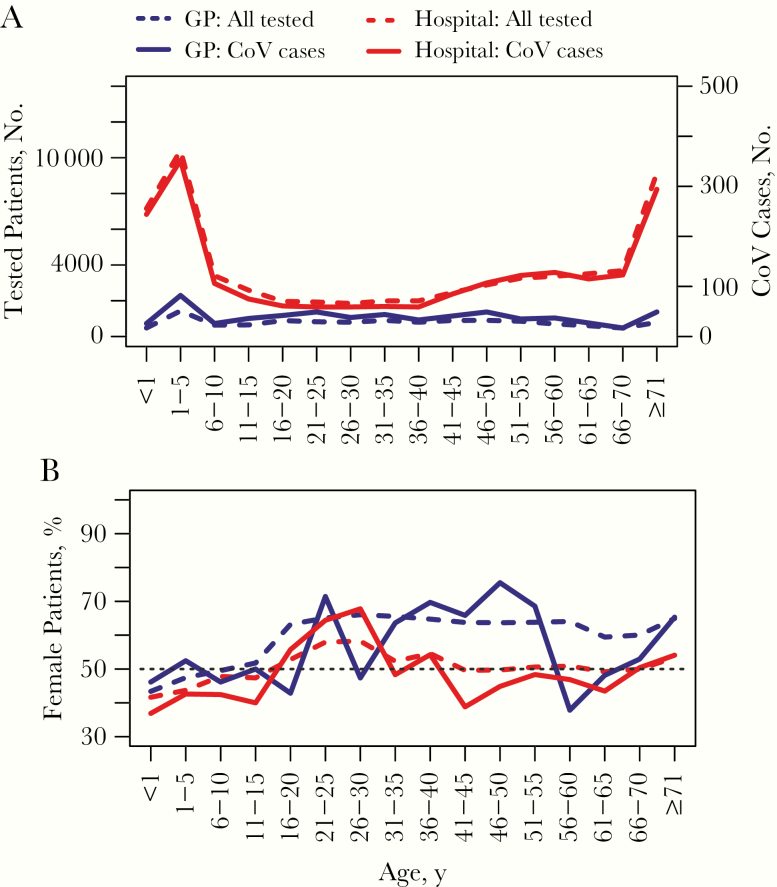
Age distributions of general practice (GP; primary care) and hospital (secondary/tertiary care) patients tested and positive for human coronavirus (CoV), (*A*) and percentages of female patients (*B*). Note the different y-axis scale for CoV cases in *A*. Hospital patients include inpatients and outpatients.

Despite more sCoV detections in the hospital setting, the prevalence was greatest among the tested GP attendees (5.3%; 673 of 12 670) than among those in hospitals (3.7%; 2285 of 61 849). Figure 3 summarizes the age distributions. Cases of sCoV in children <5 years old and the elderly (>64 years) were disproportionately represented among patients admitted to the hospital, compared with a more uniform distribution among GP attendees, closely following the overall tested population (Figure 3A). Different sex biases was found among adults depending on the healthcare setting, with more female patients in primary care versus more male patients in secondary or tertiary care (Figure 3B). This pattern was consistent when comparing the percentages of detections among sCoV-positive patients across sCoV types: 59.2% (CoV-229E), 55.6% (CoV-OC43), and 59.8% (CoV-NL63) of cases detected in primary care were in female patients, whereas 54.7% (CoV-229E), 51.1% (CoV-OC43), and 56.7% (CoV-NL63) cases detected in secondary or tertiary care were in male patients (Supplementary Table 2).

The median patient age (interquartile range) varied from 20.9 (2.7–50.2) years for CoV-NL63, to 39.9 (5.0–62.5) and 43.3 (16.5–60.4) years for CoV-OC43 and CoV-229E, respectively. The age-specific prevalences of sCoVs among the tested population are summarized in Supplementary Tables 3 and 4. More variation across ages was found in primary care patients for CoV-229E (coefficient of variation, 40.4%) and CoV-NL63 (33.8%) than for CoV-OC43 (13.6%), with less variation for patients in secondary or tertiary care (CV, 29.96% for CoV-229E, 28.0% for CoV-NL63, and 17.10% for CoV-OC43).

**Figure 4.**
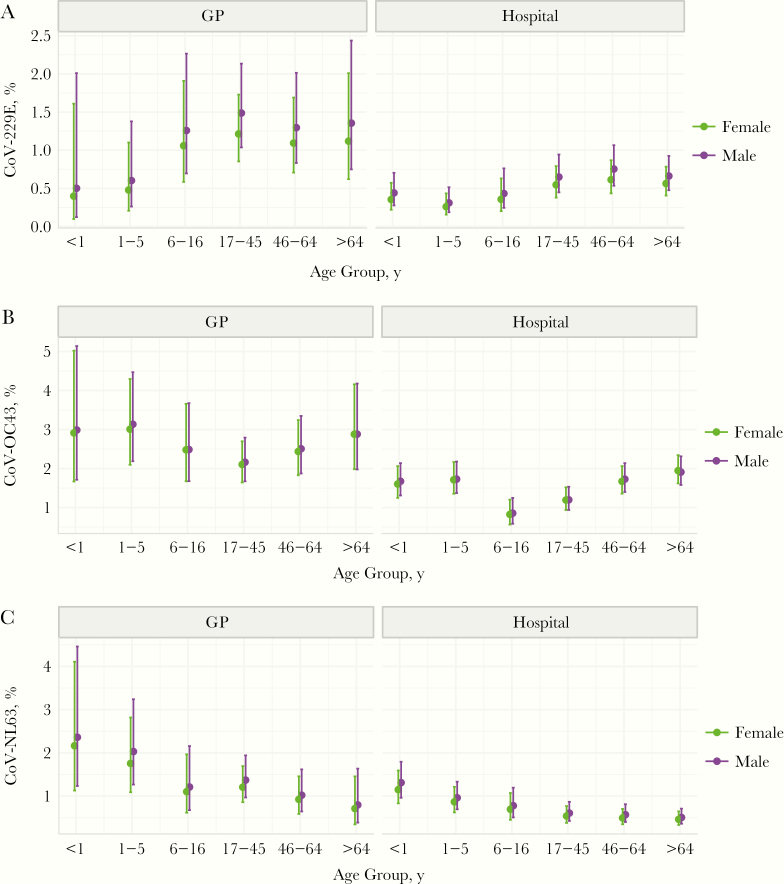
Average age-specific predicted probabilities of human coronavirus (CoV) detections by patient sex and healthcare service setting ( general practice [GP; primary care] or hospital [inpatients and outpatients; secondary or tertiary care]). Data were derived from multivariable logistic regression models incorporating statistical interactions between patient age and healthcare service (see Supplementary Tables 5–7 for model results without statistical interactions).

Statistical modeling analyses further confirmed differences in age and sex associations according to sCoV type, and a greater chance of sCoV detection among GP attendees than among patients admitted to the hospital (Supplementary Tables 5–7). No evidence of significant effect modification between patient age or sex and healthcare service setting was found (statistical interaction terms, *P* > .05; results not shown). Figure 4 summarizes average age-specific predicted probabilities with statistical interactions incorporated. In summary, we observed a trend toward increasing probability of CoV-229E with age (Figure 4A), greater probabilities of CoV-OC43 at the extremities of age (Figure 4B), and decreasing probability of CoV-NL63 with age (Figure 4C). These age patterns were broadly consistent across patient sex and healthcare settings, although we note that 95% confidence intervals overlapped across all ages except for patients in the hospital setting. A borderline significant sex effect was found for CoV-229E, with detections more likely among male patients (Supplementary Table 5).

### Variations in Seasonality Among sCoVs

**Figure 5.**
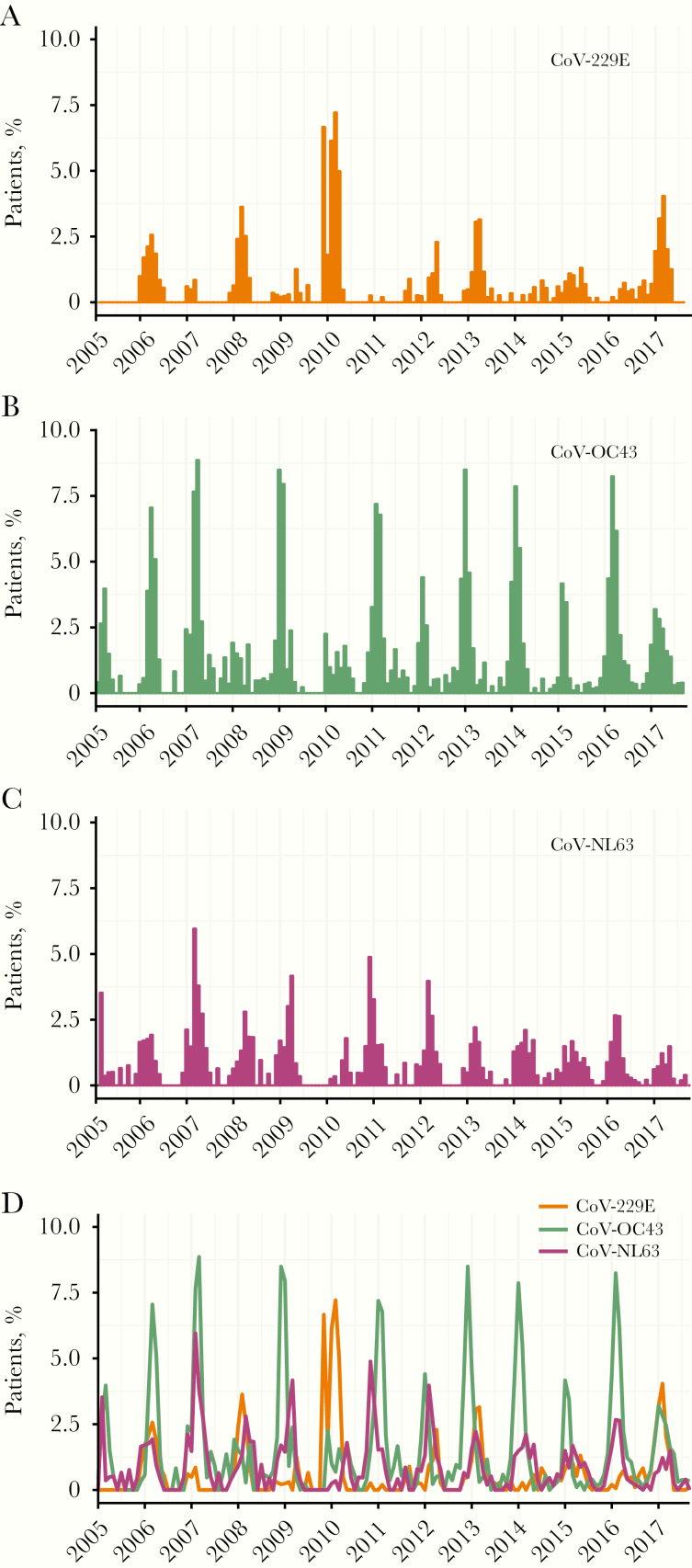
Monthly prevalence of seasonal coronaviruses (sCoVs) detected among patients with respiratory illness virologically tested in NHS Greater Glasgow and Clyde, Scotland, United Kingdom, between January 2005 and September 2017. *A,* CoV-229E. *B,* CoV-OC43. *C,* CoV-NL63. *D,* Comparing all sCoV types.

Figure 5 shows the monthly prevalences of sCoVs detected among the patient population. These are winter pathogens in the United Kingdom, peaking on average between January and March. However, there were notable variations between sCoV types and between years. Overall, CoV-OC43 was the most prevalent detection among the tested population in each influenza season. Differences were also observed in periodicities; before the first wave of pandemic influenza in 2009, CoV-229E peaked biennially, but it subsequently exhibited longer interpeak periods, particularly between 2013 and 2016.

In contrast, CoV-OC43 and CoV-NL63 generally exhibited annual periodicity of varying magnitude. A considerable degree of synchrony is observed in the timing of the peak prevalence of CoV-OC43 and CoV-NL63 for most seasons, whereas CoV-229E was more distinctive in its temporal pattern. For example, low levels of CoV-229E in 2007 coincided with high magnitudes of CoV-OC43 and CoV-NL63, whereas the high prevalence of CoV-229E in 2010 coincided with low magnitudes of CoV-OC43 and CoV-NL63.

### Interactions Between sCoVs and Other Respiratory Viruses

The cocirculation of sCoVs with other common respiratory virus raises the potential for ecological interactions, altering infection risks and the dynamics of population transmission. Our data did not permit analysis of potential within-host interactions among different sCoVs because of an absence of sCoV coinfections, but we did evaluate the potential for within-host interactions between sCoVs and other common respiratory viruses.

To do so, we analyzed the nonrandom mixing of respiratory viruses among virus-positive patients using multivariable logistic regression. We found a greater propensity for CoV-OC43 to coinfect with RSV (odds ratio, 1.68; 95% confidence interval, 1.05–2.63; uncorrected *P* = .03), AdV (2.93; 1.87–4.5, uncorrected *P* < .001), and PIV3 (2.38; 1.28–4.17; uncorrected *P* = .004) (Supplementary Table 8). The associations with AdV and PIV3 were supported after correction of *P* values for multiple comparisons (*P* < .001 and *P* = .04 respectively).

No evidence of interactions with other respiratory viruses was found for either CoV-229E or CoV-NL63. Assessment of PIV types was limited by small numbers of coinfections; these viruses were aggregated at the genus level for the CoV-229E analysis, and PIV2 was excluded from the CoV-NL63 analysis. See Supplementary Tables 9 and 10 for details and Supplementary Figure 1 for a summary. The finding for PIVB (parainfluenza virus types 2 and 4 combined; the human rubulavirus genus) must be treated with caution, because the 95% confidence interval overlaps 1. The average age-specific predicted probabilities of sCoV coinfection for individuals with or without coinfection with each specific respiratory virus are given in Supplementary Table 11.

## DISCUSSION

The likely long-term impact of the recently emerged COVID-19 is a topic currently shrouded in uncertainty for countries worldwide. At the time of writing, global cases are mounting, with evidence of community transmission for a growing number of countries. In the absence of setting-specific data, an epidemiological understanding of related and unrelated cocirculating pathogens is prudent to guide preliminary estimates of who is at risk and when, and to develop research priorities pertaining to population susceptibility. Epidemiological knowledge of sCoVs is lacking for many settings owing to an absence of inclusion in routine diagnostic testing. Here, we described several key epidemiological features of sCoVs based on a unique data set derived from multiplex PCR diagnostic testing of a large, well-defined population, over a 13-year period.

It is well known that sCoVs cocirculate endemically with other common respiratory viruses, and coinfections are frequently observed [11, 12, 16]. In the West of Scotland, sCoVs typically peak in winter months alongside influenza viruses and RSV, as described elsewhere [12]. The common occurrence of sCoVs during periods of high influenza activity highlights the importance of considering these viruses in the differential diagnosis of viral respiratory infections. This is particularly pertinent in the context of COVID-19 emergence; cocirculating viruses are associated with a broad spectrum of clinical presentation overlapping that of COVID-19, raising the potential for a large number of undiagnosed or misclassified cases in settings lacking the capacity for multiplexed testing. Currently in NHS Greater Glasgow and Clyde, Scotland, most COVID-19 testing is being conducted in the hospital setting, where severe cases of respiratory illness and high risk groups are simultaneously tested with a multiplex panel.

Sex-specific numbers of sCoV differed by healthcare setting; we observed a trend toward female patients with sCoV for primary care, but male patients for secondary or tertiary care. This finding may reflect sex differences in healthcare-seeking behaviors and/or illness severity. In terms of sex differences in detection odds (a proxy for infection and/or severity risk), a statistically significant sex effect was found for CoV-229E, with a greater odds among male than among female patients. This finding is consistent with previous reports of a male bias for CoV-OC43 and CoV-NL63 in the hospital setting [12], and in relation to influenza hospitalizations and mortality rates for acute respiratory infections more generally [23, 24]. It has been proposed that differences in sex hormones may explain variation in respiratory infection susceptibility, with testosterone exerting an immunosuppressive effect in male and estrogen playing a protective role in female [25, 26]. We note, however, that our analyses did not control for the potential confounding or effect modifying role of comorbid conditions, such as chronic obstructive pulmonary disease and asthma, or lifestyle factors, such as smoking.

A greater number of sCoV cases were observed in children <5 years of age and in elderly persons, particularly among patients admitted to hospital. This pattern is consistent with overall testing trends and may therefore reflect the healthcare-seeking behavior of concerned parents, clinician testing practices, and/or the burden of other respiratory agents, rather than a greater risk of infection. However, it should be borne in mind that these analyses were based on a patient population; the true community burden of mild and/or asymptomatic infections is likely to be much higher [27]. When aggregated into epidemiological groupings, age-specific probabilities of virus detection varied across sCoV types.

In contrast to what is generally observed for sCoVs, relatively few COVID-19 cases have been reported thus far in children [28]. In the context of our study population, COVID-19 is closely related to CoV-OC43 (both betacoronaviruses), the most prevalent sCoV detected among patients <5 years old. It is possible that preexisting cross-immunity confers protection and/or attenuates the severity of COVID-19, leading to fewer tested and hospitalized children. The comparatively lower proportion positive and detection odds for school-aged compared with younger children may potentially reflect a sustained level of CoV-OC43 immune-mediated protection, whereas waned immunity is expected to leave adults more vulnerable to CoV infection [28]. Assuming some degree of cross-immunity with sCoVs, our data are consistent with fewer expected cases of COVID-19 in children but more among the adult population [29, 30]. In addition, immunosenescence may exacerbate low levels of protective immunity in elderly persons [30, 31].

Three key features of our data seemingly support the proposition of cross-immunity. First, the contrasting age patterns of detection probabilities between closely related CoV-229E and CoV-NL63 (both alphacoronaviruses) may reflect niche segregation. Second, closely related CoV-229E and CoV-NL63 also displayed asynchronous seasonality, in contrast to CoV-OC43 and CoV-NL63, supporting a competition dynamic. Although our data did not permit in-depth analysis of CoV-HKU1, others have reported differences in the timing of peak detections with CoV-OC43 (both betacoronaviruses) [12]. Third, coinfections among sCoVs were not recorded in this study population, although detected by others albeit at a very low frequency [12]. More work is needed to establish whether low coinfection frequency among sCoVs supports an immune-mediated competition for hosts, or whether this reflects a limitation of diagnostic data that capture only a snapshot of an individual’s infection.

To our knowledge, evidence of immunological cross-protection between human CoVs is lacking, and reports of antigenic cross-reactivity are inconsistent. The potential for serological cross-reactivity between SARS-CoV and sCoVs has been shown by some [31, 32] but not others [33]. Moreover, although confinement of cross-reactivity at the CoV genus level is possible, consistent with the greater genetic relatedness of these viruses [34, 35], more general cross-reactivity between CoV-OC43 and CoV-229E has also been found [36]. Population serological surveys will be critical for establishing the true burden and age distribution of sCoV infections in the community and the potential for cross-protective immunity.

It should be borne in mind that the implications of population levels of cross-immunity are likely to vary according to the local epidemiological context. We note the predominance of CoV-OC43 detections previously observed in a comparatively urban but different Scottish patient population [14], a pattern more generally consistent at the Scottish national level [28]. Other trends observed within our study population are consistent with the Scottish national level, such as peak levels of CoV-229E detection in 2010 coinciding with low levels of CoV-NL63 and CoV-OC43 [28]. A predominance of CoV-OC43 has also been observed in Sweden [16], suggesting a potential consistency in sCoV dominance over wider geographic areas. However, differences are also apparent, for example, the relatively common detection of CoV-HKU1 in a different Scottish population [14], as also suggested in a recent report on respiratory virus detections in France [29].

Our study also highlights the potential for interactions between sCoVs and other respiratory viruses. In previous extensive analyses of virus-virus interactions, our group found a strong signal of positive interactions at the within-host scale between human CoVs overall and RSV, AdV, and PIVs (combining types 1 and 3; human *Respirovirus* genus) [6]. In the current study, our more in-depth analysis corroborates positive interactions at the sCoV type level. For CoV-OC43, the most prevalent in this study population, our results support our group’s earlier findings of a higher propensity of coinfection with RSV, AdV, or PIV3 than with other respiratory viruses. Note that these analyses were based on routine diagnostic testing, so the directionality of effects could not be determined. The association with RSV was not supported after a correction for multiple comparisons, although previous studies also showed a high proportion of sCoV coinfections with RSV [12, 37]. The role of viral coinfections in the severity of acute respiratory illness remains controversial [29–33]. Further work is needed to establish the role of viral coinfections in COVID-19 severity.

In conclusion, the public health impact of COVID-19 is likely to vary according to the epidemiological context and healthcare infrastructure of the population. Our findings suggest that continued monitoring of cocirculating respiratory viruses will be important for guiding accurate case ascertainment and research priorities surrounding population susceptibility, and for assessing the comparative population and healthcare burden of COVID-19 in the context of multiple cocirculating respiratory pathogens. Further work is needed to identify the mechanism of interactions between human CoVs and other respiratory viruses, and the role of viral coinfections in the severity and burden of COVID-19.

## Supplementary Data

Supplementary materials are available at *The Journal of Infectious Diseases* online. Consisting of data provided by the authors to benefit the reader, the posted materials are not copyedited and are the sole responsibility of the authors, so questions or comments should be addressed to the corresponding author.

jiaa185_suppl_Supplementary_Material

## Notes

***Acknowledgments.*** We are grateful to Louise Matthews, Richard Reeve, and Paul Johnson for previous helpful discussions on inferring virus-virus interactions, and we thank Beatrix von Wissmann for her critique of an earlier draft of the manuscript.

***Author contributions***. Conceptualization, data curation, methodology, formal analysis, visualization, and writing of original draft: S. N. Funding acquisition: P. R. M. Resources: D. F. P. M, J. M., and R. N. G. Investigation: S. N., A. H., D. F. P. M., and P. R. M. Manuscript review and editing: A. H., D. F. P. M., J. M., R. N. G., and P. R. M.

***Financial support.*** This work was funded by the Medical Research Council of the United Kingdom (grant MC_UU_12014/9).

***Potential conflicts of interest.*** All authors: No reported conflicts. All authors have submitted the ICMJE Form for Disclosure of Potential Conflicts of Interest. Conflicts that the editors consider relevant to the content of the manuscript have been disclosed.

## References

[1] World Health Organization. WHO announces COVID-19 outbreak a pandemic. 2020 http://www.euro.who.int/en/health-topics/health-emergencies/coronavirus-covid-19/news/news/2020/3/who-announces-covid-19-outbreak-a-pandemic. Accessed 10 April 2020.

[2] Anderson RM, Fraser C, Ghani AC, et al Epidemiology, transmission dynamics and control of SARS: the 2002–2003 epidemic. Philos Trans R Soc Lond B Biol Sci 2004; 359:1091–105. 15306395 10.1098/rstb.2004.1490 PMC1693389

[3] Mobaraki K, Ahmadzadeh J Current epidemiological status of Middle East respiratory syndrome coronavirus in the world from 1.1.2017 to 17.1.2018: a cross-sectional study. BMC Infect Dis 2019; 19:351. 31029095 10.1186/s12879-019-3987-2 PMC6487021

[4] Graham NM The epidemiology of acute respiratory infections in children and adults: a global perspective. Epidemiol Rev 1990; 12:149–78. 2286216 10.1093/oxfordjournals.epirev.a036050

[5] Van Kerkhove MD, Hirve S, Koukounari A, Mounts AW; H1N1pdm serology working group Estimating age-specific cumulative incidence for the 2009 influenza pandemic: a meta-analysis of A(H1N1)pdm09 serological studies from 19 countries. Influenza Other Respir Viruses 2013; 7:872–86. 23331969 10.1111/irv.12074 PMC5781221

[6] Nickbakhsh S, Mair C, Matthews L, et al Virus-virus interactions impact the population dynamics of influenza and the common cold. Proc Natl Acad Sci U S A 2019. 10.1073/pnas.1911083116 PMC6936719 31843887

[7] Bhattacharyya S, Gesteland PH, Korgenski K, Bjørnstad ON, Adler FR Cross-immunity between strains explains the dynamical pattern of paramyxoviruses. Proc Natl Acad Sci U S A 2015; 112:13396–400. 26460003 10.1073/pnas.1516698112 PMC4629340

[8] Casalegno JS, Ottmann M, Bouscambert-Duchamp M, Valette M, Morfin F, Lina B Impact of the 2009 influenza A(H1N1) pandemic wave on the pattern of hibernal respiratory virus epidemics, France, 2009. Euro Surveill 2010; 15:19485. 20158981

[9] Casalegno JS, Ottmann M, Duchamp MB, et al Rhinoviruses delayed the circulation of the pandemic influenza A (H1N1) 2009 virus in France. Clin Microbiol Infect 2010; 16:326–9. 20121829 10.1111/j.1469-0691.2010.03167.x

[10] Brunstein JD, Cline CL, McKinney S, Thomas E Evidence from multiplex molecular assays for complex multipathogen interactions in acute respiratory infections. J Clin Microbiol 2008; 46:97–102. 17977985 10.1128/JCM.01117-07 PMC2224244

[11] Greer RM, McErlean P, Arden KE, et al. Do rhinoviruses reduce the probability of viral co-detection during acute respiratory tract infections? J Clin Virol 2009; 45:10–5. 19376742 10.1016/j.jcv.2009.03.008 PMC7185458

[12] Gaunt ER, Hardie A, Claas EC, Simmonds P, Templeton KE Epidemiology and clinical presentations of the four human coronaviruses 229E, HKU1, NL63, and OC43 detected over 3 years using a novel multiplex real-time PCR method. J Clin Microbiol 2010; 48:2940–7. 20554810 10.1128/JCM.00636-10 PMC2916580

[13] Lepiller Q, Barth H, Lefebvre F, et al. High incidence but low burden of coronaviruses and preferential associations between respiratory viruses. J Clin Microbiol 2013; 51:3039–46. 23850954 10.1128/JCM.01078-13 PMC3754627

[14] Brittain-Long R, Andersson LM, Olofsson S, Lindh M, Westin J Seasonal variations of 15 respiratory agents illustrated by the application of a multiplex polymerase chain reaction assay. Scand J Infect Dis 2012; 44:9–17. 21867470 10.3109/00365548.2011.598876

[15] van Elden LJ, van Loon AM, van Alphen F, et al. Frequent detection of human coronaviruses in clinical specimens from patients with respiratory tract infection by use of a novel real-time reverse-transcriptase polymerase chain reaction. J Infect Dis 2004; 189:652–7. 14767819 10.1086/381207 PMC7110206

[16] Nickbakhsh S, Thorburn F, Von Wissmann B, McMENAMIN J, Gunson RN, Murcia PR Extensive multiplex PCR diagnostics reveal new insights into the epidemiology of viral respiratory infections. Epidemiol Infect 2016; 144:2064–76. 26931455 10.1017/S0950268816000339 PMC7113017

[17] Gunson RN, Bennett S, Maclean A, Carman WF Using multiplex real time PCR in order to streamline a routine diagnostic service. J Clin Virol 2008; 43:372–5. 18977692 10.1016/j.jcv.2008.08.020 PMC7108215

[18] Gunson RN, Carman WF During the summer 2009 outbreak of “swine flu” in Scotland what respiratory pathogens were diagnosed as H1N1/2009? BMC Infect Dis 2011; 11:192. 21752259 10.1186/1471-2334-11-192 PMC3146830

[19] Schwartzbaum J, Ahlbom A, Feychting M Berkson’s bias reviewed. Eur J Epidemiol 2003; 18:1109–12. 14758866 10.1023/b:ejep.0000006552.89605.c8

[20] Holm S. A simple sequentially rejective multiple test procedure. Scand J Stat 1979; 6:65–70.

[21] R Core Team. R: a language and environment for statistical computing. http://www.R-project.org/. Accessed 10 April 2020.

[22] Ludecke D ggeffects: Tidy data frames of marginal effects from regression models. J Open Source Softw 2018; 3:772.

[23] Quandelacy TM, Viboud C, Charu V, Lipsitch M, Goldstein E Age- and sex-related risk factors for influenza-associated mortality in the United States between 1997–2007. Am J Epidemiol 2014; 179:156–67. 24190951 10.1093/aje/kwt235 PMC3873104

[24] Wang XL, Yang L, Chan KH, et al. Age and sex differences in rates of influenza-associated hospitalizations in Hong Kong. Am J Epidemiol 2015; 182:335–44. 26219977 10.1093/aje/kwv068

[25] Furman D, Hejblum BP, Simon N, et al. Systems analysis of sex differences reveals an immunosuppressive role for testosterone in the response to influenza vaccination. Proc Natl Acad Sci U S A 2014; 111:869–74. 24367114 10.1073/pnas.1321060111 PMC3896147

[26] Robinson DP, Hall OJ, Nilles TL, Bream JH, Klein SL 17β-Estradiol protects females against influenza by recruiting neutrophils and increasing virus-specific CD8 T cell responses in the lungs. J Virol 2014; 88:4711–20. 24522912 10.1128/JVI.02081-13 PMC3993800

[27] Hayward AC, Fragaszy EB, Bermingham A, et al; Flu Watch Group Comparative community burden and severity of seasonal and pandemic influenza: results of the Flu Watch cohort study. Lancet Respir Med 2014; 2:445–54. 24717637 10.1016/S2213-2600(14)70034-7 PMC7164821

[28] Tang F, Quan Y, Xin ZT, et al. Lack of peripheral memory B cell responses in recovered patients with severe acute respiratory syndrome: a six-year follow-up study. J Immunol 2011; 186:7264–8. 21576510 10.4049/jimmunol.0903490

[29] Kuba K, Imai Y, Rao S, et al. A crucial role of angiotensin converting enzyme 2 (ACE2) in SARS coronavirus-induced lung injury. Nat Med 2005; 11:875–9. 16007097 10.1038/nm1267 PMC7095783

[30] Imai Y, Kuba K, Rao S, et al. Angiotensin-converting enzyme 2 protects from severe acute lung failure. Nature 2005; 436:112–6. 16001071 10.1038/nature03712 PMC7094998

[31] Ksiazek TG, Erdman D, Goldsmith CS, et al; SARS Working Group A novel coronavirus associated with severe acute respiratory syndrome. N Engl J Med 2003; 348:1953–66. 12690092 10.1056/NEJMoa030781

[32] Sun ZF, Meng XJ Antigenic cross-reactivity between the nucleocapsid protein of severe acute respiratory syndrome (SARS) coronavirus and polyclonal antisera of antigenic group I animal coronaviruses: implication for SARS diagnosis. J Clin Microbiol 2004; 42:2351–2. 15131233 10.1128/JCM.42.5.2351-2352.2004 PMC404591

[33] Peiris JS, Lai ST, Poon LL, et al; SARS study group Coronavirus as a possible cause of severe acute respiratory syndrome. Lancet 2003; 361:1319–25. 12711465 10.1016/S0140-6736(03)13077-2 PMC7112372

[34] Wang N, Li SY, Yang XL, et al. Serological evidence of bat SARS-related coronavirus infection in humans, China. Virol Sin 2018; 33:104–7. 29500691 10.1007/s12250-018-0012-7 PMC6178078

[35] Lehmann C, Wolf H, Xu J, et al. A line immunoassay utilizing recombinant nucleocapsid proteins for detection of antibodies to human coronaviruses. Diagn Microbiol Infect Dis 2008; 61:40–8. 18191362 10.1016/j.diagmicrobio.2007.12.002 PMC7127592

[36] Chan KH, Cheng VC, Woo PC, et al. Serological responses in patients with severe acute respiratory syndrome coronavirus infection and cross-reactivity with human coronaviruses 229E, OC43, and NL63. Clin Diagn Lab Immunol 2005; 12:1317–21. 16275947 10.1128/CDLI.12.11.1317-1321.2005 PMC1287763

[37] Nolan VG, Arnold SR, Bramley AM, et al. Etiology and impact of coinfections in children hospitalized with community-acquired pneumonia. J Infect Dis 2018; 218:179–88. 29228381 10.1093/infdis/jix641 PMC7108488

