# Supplementary material for: Epidemiology of Seasonal Coronaviruses: Establishing the Context for the Emergence of Coronavirus Disease 2019
Source: J Infect Dis. 2020 Apr 14;222(1):17–25. doi: 10.1093/infdis/jiaa185 (PMC7184404; doi:10.1093/infdis/jiaa185)
Supplement: jiaa185_suppl_Supplementary_Material [file jiaa185_suppl_supplementary_material.pdf]

Supplementary Material

Epidemiology of Seasonal Coronaviruses: Establishing the Context for the Emergence of Coronavirus Disease 2019

Sema Nickbakhsh[a*], Antonia Ho[a], Diogo F.P. Marques[b], Jim McMenamin[b], Rory N. Gunson[c], Pablo R. Murcia[a]

[a]MRC-University of Glasgow Centre for Virus Research, Institute of Infection, Immunity and Inflammation, College of Medical, Veterinary and Life Sciences, University of Glasgow, Glasgow, United Kingdom.

[b]Public Health Scotland, NHS National Services Scotland, G2 6QE Glasgow, United Kingdom.

[c]West of Scotland Specialist Virology Centre, NHS Greater Glasgow and Clyde, Glasgow, United Kingdom.

***Corresponding author:** Sema Nickbakhsh, MRC-University of Glasgow Centre for Virus Research, 646 Bearsden Road, Glasgow, G61 1QH, email: sema.nickbakhsh@glasgow.ac.uk

Table S1: Coronavirus detection frequencies among 68,819 patients tested in periods pre and post major waves of H1N1 influenza A pandemic virus circulation, NHS Greater Glasgow and Clyde, Scotland, UK.

**Measure of seasonal coronavirus (sCoV) detection frequency**	**Pre-pandemic Jan 2005 – Apr 2009**	**Post- pandemic Mar 2011 – Sep 2017**
Total sCoV detections	545[*] (13.34[a]; 4.27[b])	2072[*] (9.55[a]; 3.70[b])
CoV-229E detections (% among sCoV-positives)	78 (14.31)	382 (18.44)
CoV-OC43 detections (% among sCoV-positives)	267 (49.0)	1063 (51.30)
CoV-NL63 detections (% among sCoV-positives)	164 (30.09)	474 (22.88)
Total number of virus-positive patients[†]	4,086	21,690
Total number of tested patients	12,755	56,064

[*]Note: a small number (n=278; 9.4% out of 74,519 total episodes) of CoV detections were not typed so individual sCoVs do not sum to total; [a] % among total positive; [b] % among total tested. [†]Patients positive to at least one of influenza A, influenza B, respiratory syncytial virus, human coronavirus, human metapneumovirus, adenovirus, rhinovirus, parainfluenza types 1-4.

Table S2: Distribution of coronavirus detections by patient sex (%): among patients positive for CoV-229E, CoV-OC43 and CoV-NL63, NHS Greater Glasgow and Clyde, Scotland, UK, 2005-2017

Healthcare service setting	Seasonal coronavirus type	Female	Male
General Practice (primary care)	CoV-229E	87 (59.2)	60 (40.8)
	CoV-OC43	175 (55.6)	140 (44.4)
	CoV-NL63	101 (59.8)	68 (40.2)
Hospital (secondary care)	CoV-229E	175 (44.3)	211 (54.7)
	CoV-OC43	553 (48.9)	577 (51.1)
	CoV-NL63	231 (43.3)	302 (56.7)

Table S3: Age distribution of coronavirus detections (%): among 12,628 patients tested for CoV-229E, CoV-OC43 and CoV-NL63 in primary care, NHS Greater Glasgow and Clyde, Scotland, UK, 2005-2017

Age group	CoV-229E	CoV-OC43	CoV-NL63	Total positive	Total tested
<1y	2 (0.42)	14 (2.94)	10 (2.10)	26	477
1-5y	8 (0.56)	44 (3.07)	30 (2.09)	82	1433
6-16y	17 (1.18)	34 (2.35)	18 (1.24)	69	1446
17-46y	69 (1.39)	107 (2.16)	64 (1.29)	240	4950
47-64y	34 (1.15)	76 (2.58)	34 (1.15)	144	2947
≥65y	17 (1.24)	40 (2.91)	13 (0.95)	70	1375
Total	147 (1.16)	315 (2.49)	169 (1.34)	631	12628

Table S4: Age distribution of coronavirus detections (%): among 61,613 patients tested for CoV-229E, CoV-OC43 and CoV-NL63 in secondary care, NHS Greater Glasgow and Clyde, Scotland, UK, 2005-2017

Age group	CoV-229E	CoV-OC43	CoV-NL63	Total positive	Total tested
<1y	30 (0.42)	127 (1.77)	87 (1.22)	244	7159
1-5y	39 (0.37)	202 (1.94)	111 (1.07)	352	10408
6-16y	39 (0.61)	81 (1.26)	72 (1.12)	192	6405
17-46y	81 (0.69)	190 (1.61)	101 (0.86)	372	11779
47-64y	106 (0.86)	252 (2.04)	86 (0.70)	444	12341
≥65y	91 (0.67)	278 (2.06)	76 (0.56)	445	13521
Total	386 (0.63)	1130 (1.83)	533 (0.90)	2049	61613

Table S5: Multivariable logistic regression analysis of factors associated with CoV-229E detections

Explanatory variable	Levels	Odds ratio	95% CI	p-value
Age group	1-5y (Reference)	1	-	-
	<1y	1.39	0.81-2.41	0.228
	6-16y	1.55	0.9-2.67	0.11
	17-45y	2.12	1.39-3.34	0.001[*]
	46-64y	2.19	1.44-3.47	<0.001[*]
	≥65y	2.06	1.34-3.27	0.002[*]
Sex	Female (Reference)	1	-	-
	Male	1.23	1-1.52	0.055[*]
Healthcare setting[†]	GP (Reference)	1	-	-
	Hospital	0.57	0.46-0.73	<0.001[*]
Time period[‡]	Pre-pandemic (Reference)	1	-	-
	Pandemic	2.28	1.58-3.28	<0.001[*]
	Post-pandemic	1.11	0.84-1.5	0.477
Season	Dec – Feb (winter) (Reference)	1	-	-
	Sep – Nov (autumn)	0.29	0.2-0.42	<0.001[*]
	Mar – May (spring)	0.82	0.65-1.04	0.102
	Jun – Aug (summer)	0.19	0.11-0.29	<0.001[*]

[*]Significant or borderline significant at 5% alpha-level. CI=confidence interval. [†]GP: general practice; Hospital: inpatients and outpatients; [‡]Pre-pandemic: January 2005 – April 2009, pandemic: May 2009 – February 2011, post-pandemic: March 2011 – September 2017.

Table S6: Multivariable logistic regression analysis of factors associated with CoV-OC43 detections

Explanatory variable	Levels	Odds ratio	95% CI	p-value
Age group	1-5y (Reference)	1	-	-
	<1y	1.01	0.79-1.29	0.95
	6-16y	0.55	0.4-0.74	<0.001[*]
	17-45y	0.66	0.53-0.83	<0.001[*]
	46-64y	0.87	0.71-1.08	0.21
	≥65y	1	0.82-1.23	0.981
Sex	Female (Reference)	1	-	-
	Male	1.04	0.91-1.18	0.562
Healthcare setting[†]	GP (Reference)	1	-	-
	Hospital	0.7	0.6-0.81	<0.001[*]
Time period[‡]	Pre-pandemic (Reference)	1	-	-
	Pandemic	1.05	0.81-1.34	0.708
	Post-pandemic	0.96	0.82-1.14	0.656
Season	Dec – Feb (winter) (Reference)	1	-	-
	Sep – Nov (autumn)	0.15	0.12-0.2	<0.001[*]
	Mar – May (spring)	0.49	0.43-0.57	<0.001[*]
	Jun – Aug (summer)	0.09	0.07-0.13	<0.001[*]

[*]Significant at 5% alpha-level. CI=confidence interval. [†]GP: general practice; Hospital: inpatients and outpatients; [‡]Pre-pandemic: January 2005 – April 2009, pandemic: May 2009 – February 2011, post-pandemic: March 2011 – September 2017.

Table S7: Multivariable logistic regression analysis of factors associated with CoV-NL63 detections

Explanatory variable	Levels	Odds ratio	95% CI	p-value
Age group	1-5y (Reference)	1	-	-
	<1y	1.41	1.04-1.91	0.026[*]
	6-16y	0.69	0.47-0.99	0.05[*]
	17-45y	0.63	0.47-0.85	0.003[*]
	46-64y	0.54	0.39-0.74	<0.001[*]
	≥65y	0.48	0.34-0.67	<0.001[*]
Sex	Female (Reference)	1	-	-
	Male	1.14	0.94-1.37	0.182
Healthcare setting[†]	GP (Reference)	1	-	-
	Hospital	0.59	0.47-0.73	<0.001[*]
Time period[‡]	Pre-pandemic (Reference)	1	-	-
	Pandemic	1.13	0.8-1.58	0.469
	Post-pandemic	0.8	0.64-1.01	0.056[*]
Season	Dec – Feb (winter) (Reference)	1	-	-
	Sep – Nov (autumn)	0.21	0.14-0.31	<0.001[*]
	Mar – May (spring)	1.05	0.86-1.29	0.614
	Jun – Aug (summer)	0.18	0.11-0.27	<0.001[*]

[*]Significant or borderline significant at 5% alpha-level; CI=confidence interval. [†]GP: general practice; Hospital: inpatients and outpatients; [‡]Pre-pandemic: January 2005 – April 2009, pandemic: May 2009 – February 2011, post-pandemic: March 2011 – September 2017.

Table S8: Investigating viruses interacting with CoV-OC43 using multivariable binary logistic regression (AUC=79.6%, 95% CI=76.46%-82.75%)

Explanatory variable	Levels	Odds ratio	95% CI	p-value
Age group	1-5y (Reference)	1	-	-
	<1y	1.27	0.85-1.88	0.236
	6-16y	0.55	0.26-1.06	0.093
	17-45y	0.7	0.42-1.14	0.161
	46-64y	0.74	0.43-1.23	0.256
	>64y	1.09	0.68-1.71	0.723
Sex	Female (Reference)	1	-	-
	Male	1.77	1.32-2.39	<0.001
Healthcare setting*	GP (Reference)	1	-	-
	Hospital	0.96	0.67-1.43	0.853
Time period†	Pre-pandemic (Reference)	1	-	-
	Pandemic	1.05	0.56-1.9	0.874
	Post-pandemic	0.78	0.46-1.31	0.34
CoV-OC43 infection frequency‡	-	2.91	2.41-3.54	<0.001
CoV-OC43 testing frequency‡	-	0.47	0.33-0.66	<0.001
IAV infection	No (Reference)	1	-	-
	Yes	0.9	0.52-1.5	0.682
IBV infection	No (Reference)	1	-	-
	Yes	0.93	0.44-1.83	0.849
RV infection	No (Reference)	1	-	-
	Yes	1.1	0.72-1.65	0.653
RSV infection	No (Reference)	1	-	-
	Yes	1.68	1.05-2.63	0.027[a]
MPV infection	No (Reference)	1	-	-
	Yes	0.84	0.39-1.62	0.624
AdV infection	No (Reference)	1	-	-
	Yes	2.93	1.87-4.5	<0.001[b]
PIV1 infection	No (Reference)	1	-	-
	Yes	0.71	0.04-3.34	0.741
PIV2 infection	No (Reference)	1		
	Yes	0.91	0.05-4.31	0.93
PIV3 infection	No (Reference)	1		
	Yes	2.38	1.28-4.17	0.004[b]
PIV4 infection	No (Reference)	1	-	-
	Yes	0.73	0.12-2.38	0.664

[a] Significant at 5% alpha-level pre-correction for multiple comparisons. [b] Significance upheld following Holm's correction for multiple comparisons: AdV corrected p<0.001; PIV3 corrected p=0.036. CI=confidence interval. *GP: general practice; Hospital: inpatients and outpatients; †Pre-pandemic: January 2005 - April 2009; pandemic: May 2009 - February 2011; post-pandemic: March 2011 - September 2017; ‡Infection and testing frequencies adjusting for response virus seasonality; IAV=influenza A virus [H1N1 and H3N2]; IBV=influenza B virus; RV=human rhinoviruses; MPV=human metapneumovirus; AdV=adenoviruses; PIV=parainfluenza virus (types 1-4).

Table S9: Investigating viruses interacting with CoV-229E using multivariable binary logistic regression (AUC=79.90%, 95% CI=74.32%-85.48%)

Explanatory variable	Levels	Odds ratio	95% CI	p-value
Age group	1-5y (Reference)	1	-	-
	<1y	0.86	0.35-2.04	0.741
	6-16y	1.32	0.41-3.6	0.61
	17-45y	1.55	0.69-3.52	0.286
	46-64y	2.39	1.13-5.23	0.024
	$\geq$65y	1.59	0.7-3.65	0.269
Sex	Female (Reference)	1	-	-
	Male	1.06	0.66-1.68	0.819
Healthcare setting*	GP (Reference)	1	-	-
	Hospital	1	0.57-1.85	0.996
Time period†	Pre-pandemic (Reference)	1	-	-
	Pandemic	1.08	0.42-2.77	0.873
	Post-pandemic	0.63	0.25-1.61	0.321
CoV-229E infection frequency‡	-	3.1	2.35-4.17	<0.001
CoV-229E testing frequency‡	-	0.6	0.36-1.01	0.052
IAV infection	No (Reference)	1	-	-
	Yes	1.23	0.49-2.83	0.641
IBV infection	No (Reference)	1	-	-
	Yes	0.63	0.16-1.93	0.459
RV infection	No (Reference)	1	-	-
	Yes	1.22	0.55-2.49	0.606
RSV infection	No (Reference)	1	-	-
	Yes	2.12	0.89-4.58	0.069
MPV infection	No (Reference)	1	-	-
	Yes	1.78	0.65-4.31	0.227
AdV infection	No (Reference)	1	-	-
	Yes	1.77	0.71-3.96	0.188
PIVA infection	No (Reference)	1	-	-
	Yes	1.5	0.46-3.95	0.455
PIVB infection	No (Reference)	1		
	Yes	3.34	0.93-9.09	0.034[a]

[a] Result does not support significance; note 95% CI overlaps 1. *GP: general practice; Hospital: inpatients and outpatients; †Pre-pandemic: January 2005 - April 2009; pandemic: May 2009 - February 2011; post-pandemic: March 2011 - September 2017; ‡Infection and testing frequencies adjusting for response virus seasonality; IAV=influenza A virus [H1N1 and H3N2]; IBV=influenza B virus; RV=human rhinoviruses; MPV=human metapneumovirus; AdV=adenoviruses; PIVA=parainfluenza virus (PIV) types 1 and 3 (human respirovirus genus); and PIVB=PIV2 and PIV4 (human rubulavirus genus).

Table S10: Investigating viruses interacting with CoV-NL63 using multivariable binary logistic regression (AUC=79.95%, 95% CI=75.29%-84.61%)

Explanatory variable	Levels	Odds ratio	95% CI	p-value
Age group	1-5y (Reference)	1	-	-
	<1y	1.6	0.96-2.67	0.072
	6-16y	0.5	0.17-1.21	0.159
	17-45y	0.51	0.23-1.05	0.078
	46-64y	0.29	0.1-0.69	0.011
	>64y	0.11	0.02-0.39	0.003
Sex	Female (Reference)	1	-	-
	Male	0.74	0.48-1.13	0.162
Healthcare setting[*]	GP (Reference)	1	-	-
	Hospital	0.93	0.54-1.71	0.805
Time period[†]	Pre-pandemic (Reference)	1	-	-
	Pandemic	0.61	0.26-1.29	0.215
	Post-pandemic	0.37	0.17-0.78	0.01
CoV-NL63 infection frequency[‡]	-	2.58	1.88-3.59	<0.001
CoV-NL63 testing frequency[‡]	-	0.69	0.42-1.15	0.151
IAV infection	No (Reference)	1	-	-
	Yes	0.49	0.17-1.23	0.154
IBV infection	No (Reference)	1	-	-
	Yes	0.67	0.2-1.89	0.478
RV infection	No (Reference)	1	-	-
	Yes	1.03	0.53-1.92	0.916
RSV infection	No (Reference)	1	-	-
	Yes	1.23	0.58-2.45	0.566
MPV infection	No (Reference)	1	-	-
	Yes	0.82	0.26-2.08	0.698
AdV infection	No (Reference)	1	-	-
	Yes	1.26	0.61-2.44	0.507
PIV1 infection	No (Reference)	1	-	-
	Yes	1.26	0.07-6.15	0.823
PIV3 infection	No (Reference)	1	-	-
	Yes	1.52	0.6-3.4	0.339
PIV4 infection	No (Reference)	1	-	-
	Yes	0.73	0.04-3.57	0.764

CI=confidence interval. [*]GP: general practice; Hospital: inpatients and outpatients; [†]Pre-pandemic: January 2005 - April 2009; pandemic: May 2009 - February 2011; post-pandemic: March 2011 - September 2017; [‡]Infection and testing frequencies adjusting for response virus seasonality; IAV=influenza A virus [H1N1 and H3N2]; IBV=influenza B virus; RV=human rhinoviruses; MPV=human metapneumovirus; AdV=adenoviruses; PIV=parainfluenza virus (types 1,3 or 4). Note parainfluenza type 2 was excluded from this analysis due to zero observed coinfections.

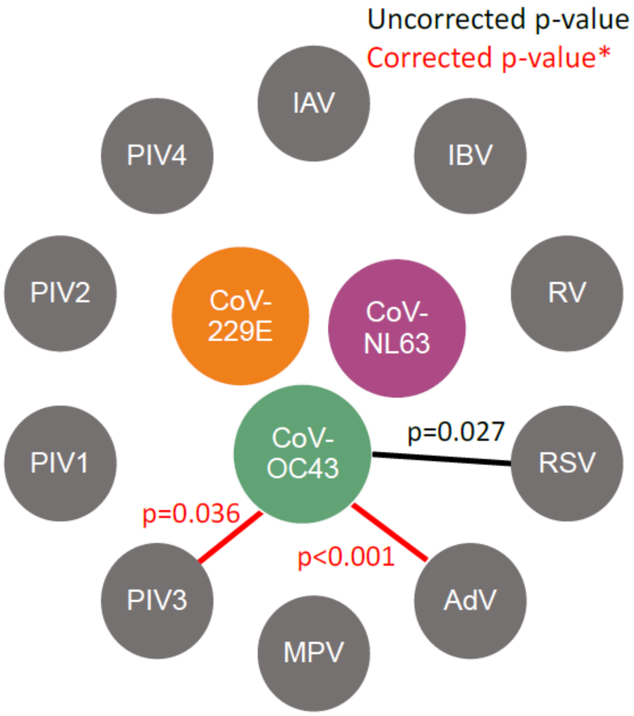

Numbers of coinfections of seasonal coronaviruses with other common respiratory viruses detected among 16,991 virus-positive patients informing the statistical regression analyses.

	CoV-OC43	CoV-NL63	CoV-229E
**RV**	67	37	23
**RSV**	46	19	16
**IAV**	36	7	15
**AdV**	48	16	9
**IBV**	12	5	4
**PIV3**	16	8	3
**MPV**	10	5	8
**PIV4**	2	1	3
**PIV1**	1	1	2
**PIV2**	1	0	1
**Total**	**239**	**99**	**84**

* Note viruses are listed in descending order of their overall prevalence in the patient population. No coinfections were observed among seasonal coronavirus types.

Figure S1: Interactions between human coronaviruses (CoV) and other respiratory viruses inferred using multivariable logistic regression based on 16,991 respiratory virus-positive patients. See supplementary Tables S8-S10 for full results. *p-values corrected for multiple comparisons using Holm's method. CoV=human coronavirus; IAV=influenza A virus (H1N1 and H3N2); IBV=influenza B virus; RV=human rhinoviruses; RSV=respiratory syncytial virus; AdV=human adenoviruses; MPV=human metapneumovirus; PIV1=parainfluenza 1 virus; PIV2=parainfluenza 2 virus; PIV3=parainfluenza 3 virus; PIV4=parainfluenza 4 virus. Note PIV types were aggregated into PIV1/PIV3 and PIV2/PIV4 (genus groups) for the CoV-229E analysis, and PIV2 was excluded from the CoV-NL63 analysis.

Table S11: Age-specific predicted probabilities of CoV-OC43 detection among patients with and without putative interacting viruses

Interacting virus	PCR-positive	Age group	Predicted probability*	Lower 95% CI†	Upper 95% CI†
AdV	No	<1y	0.014	0.008	0.025
	Yes		0.038	0.021	0.067
	No	1-5y	0.011	0.006	0.020
	Yes		0.031	0.018	0.054
	No	6-16y	0.006	0.003	0.014
	Yes		0.017	0.007	0.036
	No	17-45y	0.008	0.004	0.015
	Yes		0.018	0.010	0.035
	No	46-64y	0.008	0.004	0.016
	Yes		0.020	0.010	0.042
	No	>64y	0.012	0.007	0.022
	Yes		0.030	0.017	0.054
PIV3	No	<1y	0.017	0.010	0.029
	Yes		0.021	0.011	0.041
	No	1-5y	0.015	0.009	0.026
	Yes		0.020	0.010	0.039
	No	6-16y	0.007	0.003	0.017
	Yes		0.008	0.003	0.019
	No	17-45y	0.008	0.004	0.016
	Yes		0.010	0.005	0.021
	No	46-64y	0.008	0.004	0.016
	Yes		0.009	0.004	0.019
	No	>64y	0.012	0.007	0.022
	Yes		0.013	0.007	0.026

* Predicted probability generated from fitted multivariable logistic regression models based on 16,991 respiratory virus-positive patients. See supplementary Tables S8-S10 for full analysis results. †CI=confidence interval.
